# Supplementary material for: Global hypo-methylation in a proportion of glioblastoma enriched for an astrocytic signature is associated with increased invasion and altered immune landscape
Source: eLife. 2022 Nov 22;11:e77335. doi: 10.7554/eLife.77335 (PMC9681209; doi:10.7554/eLife.77335)
Supplement: Figure 2—source data 1. [file elife-77335-fig2-data1.zip › Figure_2_source_data_1/Figure_2A/homerResults/motif27.similar.html]

motif27

## Information for motif27

A
G
T
C
A
G
T
C
A
G
T
C
A
G
C
T
A
C
T
G
A
C
T
G
A
C
T
G
A
C
T
G
C
G
T
A
A
C
T
G
  
Reverse Opposite:  

A
G
T
C
A
C
G
T
A
G
T
C
A
G
T
C
A
G
T
C
A
G
T
C
C
T
G
A
C
T
A
G
A
C
T
G
A
C
T
G
  

|  |  |
| --- | --- |
| p-value: | 1e-17 |
| log p-value: | -4.124e+01 |
| Information Content per bp: | 1.946 |
| Number of Target Sequences with motif | 24.0 |
| Percentage of Target Sequences with motif | 1.03% |
| Number of Background Sequences with motif | 3.5 |
| Percentage of Background Sequences with motif | 0.10% |
| Average Position of motif in Targets | 113.4 +/- 48.7bp |
| Average Position of motif in Background | 66.6 +/- 6.3bp |
| Strand Bias (log2 ratio + to - strand density) | -0.3 |
| Multiplicity (# of sites on avg that occur together) | 1.04 |
| Motif File: | file (matrix) reverse opposite |

### Similar de novo motifs found

|  |  |  |  |  |  |  |  |
| --- | --- | --- | --- | --- | --- | --- | --- |
| Rank | Match Score | Redundant Motif | P-value | log P-value | % of Targets | % of Background | Motif file |
| 1 | 0.612 | A C G T T A G C G A T C T C G A A C G T A T C G T A C G A C T G T A G C G C T A A T G C T G A C | 1e-12 | -28.956684 | 0.82% | 0.08% | motif file (matrix) |
| 2 | 0.721 | A G T C A C G T A G T C A C T G A T C G A C T G C G T A A C T G | 1e-7 | -17.570472 | 0.82% | 0.19% | motif file (matrix) |
| 3 | 0.666 | A G T C C G T A A G T C A C G T A G T C A G T C A G T C A G T C C G T A A G T C | 1e-5 | -12.263405 | 0.30% | 0.03% | motif file (matrix) |
